# Gastrin and the Moderate Hypergastrinemias

**DOI:** 10.3390/ijms22136977

**Published:** 2021-06-29

**Authors:** Jens F. Rehfeld

**Affiliations:** Department of Clinical Biochemistry, Rigshospitalet, University of Copenhagen, DK-2100 Copenhagen, Denmark; jens.f.rehfeld@regionh.dk

**Keywords:** duodenal ulcer, gastric cancer, gastrin, *Helicobacter pylori*, hypergastrinemia, proton-pump inhibitors

## Abstract

The antral hormone gastrin potently regulates gastric acid secretion and fundic mucosal growth. Consequently, appropriate gastrin secretion and plasma concentrations are important for the early phases of digestion. This review describes as the first premise the normal biogenesis of gastrin in the antral mucosa, but also mentions the extraantral expression. Subsequently, the molecular nature and concentration levels of gastrin in serum or plasma are overviewed. Third, assays for accurate measurements of plasma or serum concentrations are commented. Finally, the problem of moderate hypergastrinemia due to *Helicobacter pylori* infections and/or treatment with proton-pump inhibitors (PPI) is discussed. The review concludes that accurate measurement of the true concentrations of bioactive gastrins in plasma is important. Moreover, it suggests that moderate hypergastrinemias are also essential health issues that require serious attention.

## 1. Introduction

**Figure 1:**
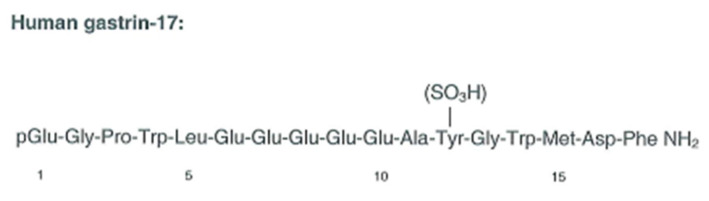
The structure of gastrin-17, the first identified molecular form of gastrin in antral G cells [15].

Gastrin is the gastroduodenal hormone that stimulates acid secretion and growth of the fundic mucosa (see Figure 1 and the recent reviews [1,2]). Precisely regulated gastrin secretion is essential for normal digestion and, consequently, for good health. In contrast, either total lack of gastrin or the opposite, severe hypersecretion of gastrin, are life-threatening. Complete lack of gastrin is seen in genetically modified “knockout” mice. Such mice cannot produce gastric acid [3,4]. However, gastric acid is necessary for killing ingested bacteria and other pathogenic microorganisms. In the absence of gastric acid, the stomach, therefore, becomes infected with bacteria that gradually lead to irreversible intestinal metaplasia and carcinogenic tumor development [5,6]. Vice versa, severe hypersecretion of gastrin as seen in the Zollinger–Ellison syndrome caused by gastrin-producing tumors (gastrinomas) is, in its fulminant forms, also life-threatening—partly due to gross gastric hyperacidity with multiple duodenal and jejunal peptic ulcers, diarrhea and severe water–electrolyte disturbances and partly because gastrinomas—although mostly slow-growing—are malignant, metastatic neoplasias [7,8]. In addition to the relatively rare neuroendocrine gastrinomas, however, gastrin is also expressed locally in common cancers, for instance, in some brain tumors, most lung cancers, exocrine pancreatic cancers, gastric, colorectal and ovarian adenocarcinomas in which autocrine and/or paracrine gastrin may stimulate carcinogenetic growth [9,10,11,12,13,14].

In between the described extremes of gastrin secretion—from complete lack to excessive overproduction—are the moderate variations of plasma gastrin levels (Table 1). In addition to the variations of fasted and fed normogastrinemic healthy persons, they include hypogastrinemias as seen, for instance, after classic Whipple operations (where the antrum, the duodenum, the upper jejunum and the head of the pancreas have been removed [16]) and in achlorhydric patients with pernicious anemia also involving atrophy of the antral mucosa [17]. Frequent moderate hypergastrinemias are, however, seen in *Helicobacter pylori*-infected patients [18,19,20,21,22,23,24] and in patients who are treated with proton-pump inhibitors [25,26,27,28,29,30,31,32,33].

This review discusses, in particular, moderate hypergastrinemias and their possible pathogenetic significance. In order to have such a discussion, an essential premise is first to understand the biogenesis of bioactive gastrin peptides in antral G cells, and then to also consider the cellular expression of gastrin outside the antral mucosa.

## 2. G Cell Synthesis of Gastrin

**Figure 2:**
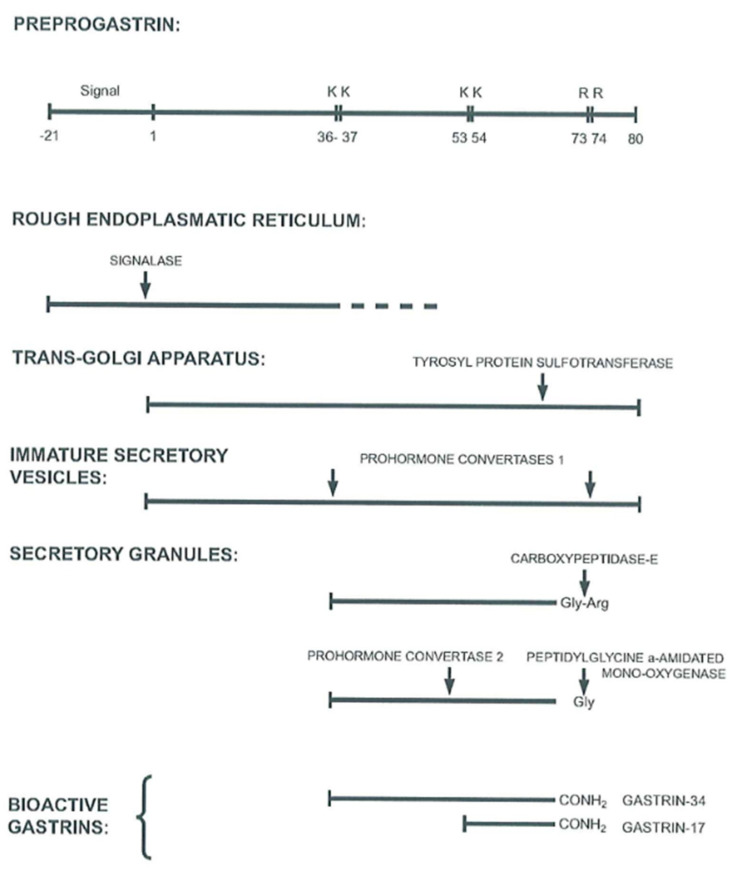
Scheme of processing steps for progastrin to release the two major bioactive gastrins, gastrin-17 and gastrin-34. Both forms are synthetized partly tyrosyl-sulfated. The endoproteolytic cleavages of progastrin are performed by prohormone convertases 1/3 and 2 [37,39].

In healthy adult humans, G cells in the antral and duodenal mucosa are the main site of gastrin synthesis and subsequent release to blood (for a recent review see [2]). A few sporadic G cells are also present in the jejunum and in the ileum. So far, however, gastrin biosynthesis studies have examined antral gastrin production [34,35,36]. Interpretation of the results of these studies in the light of general knowledge about peptide biosynthesis provides a picture of normal synthesis of antroduodenal gastrin, as shown in Figure 2 and as detailed earlier [37,38].

After translation of gastrin mRNA in the rough endoplasmic reticulum and cotranslational removal of the signal peptide from preprogastrin, progastrin is transported to the Golgi apparatus where the first posttranslational modifications occur (Figure 2). These are *O*-sulfation by sulfotransferases of the Tyr66 residue and the first endoproteolytic cleavages by prohormone convertase 1/3 at the dibasic Arg36Arg37 and Arg73Arg74 sites. The dibasic Lys53Lys54 site in the middle of the gastrin-34 sequence is cleaved later by prohormone convertase 2 [39]. From the trans-Golgi network, secretory vesicles carry their cargo of processing intermediates toward the basal part of G cells where the peptides are stored and condensed in secretory granules.

The endoproteolytic processing and exoproteolytic trimming, such as removal of the Arg73Arg74 residues by carboxypeptidase E, as well as the subsequent glutamyl cyclization of the N-termini of gastrin-34 and gastrin-17, continue during the transport to the early secretory granules. The last and decisive processing step in the synthesis then occurs during storage in the maturing secretory granules, where the peptide *α*-amidation enzyme complex (PAM) removes glyoxylate from the C-terminal glycyl residue in the immediate precursors, the glycine-extended gastrins [40]. Partial phosphorylation of serine in the C-terminal flanking fragment may also occur, but the significance of this modification and nature of the kinase involved are not yet known [41].

**Figure 3:**
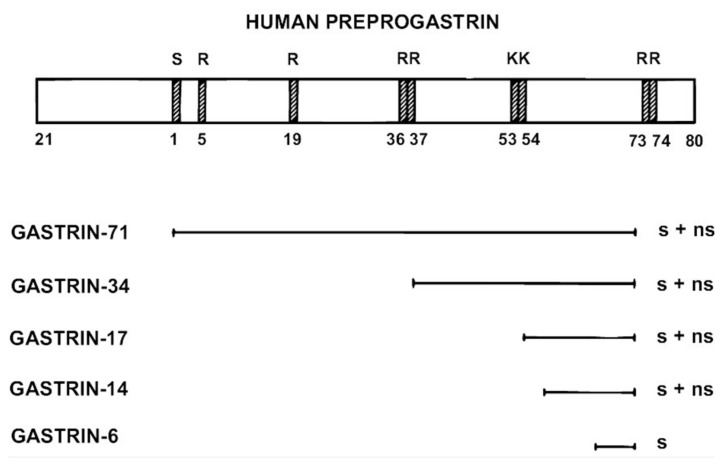
Scheme of carboxyamidated products of preprogastrin. Endoproteolytic cleavage sites (R and K) are shown on preprogastrin. S and ns mean “sulfated” and “non-sulfated”, respectively.

As a result of multiple steps in the biosynthetic pathway, of which none are complete (sic!), G cells release a heterogeneous mixture of progastrin products from the secretory granules to the surrounding capillaries. A small percentage comprises non-amidated precursors and processing intermediates, including the glycine-extended gastrins. However, in a normal human, more than 90% are *α*-amidated bioactive gastrins, the longest form of which is gastrin-71 [42]. Of the amidated gastrins, around 85% are gastrin-17, 10% are gastrin-34 and the rest are a mixture of gastrin-71, a little gastrin-52, some gastrin-14 and a short sulfated C-terminal hexapeptide amide, gastrin-6 [43] (Figure 3).

Of significance for understanding hypergastrinemic conditions is the realization that the faster rate of gastrin biosynthesis changes the molecular pattern in gastrin-producing tissues. Such increased synthesis occurs in humans—as mentioned—in gastrinomas, occasionally in gastric juvenile polyposis [44], as well as in *Helicobacter pylori* infections in the antral mucosa, achlorhydria as seen in pernicious anemia and during PPI therapy. In these disorders, the biosynthesis in G cells is increased and the intracellular transport of secretory vesicles is enhanced so that processing enzymes apparently cannot keep up with the processing of progastrin. Consequently, gastrinoma cells, gastric polyposis cells and antral G cells in achlorhydric and *Helicobacter*-infected stomachs release larger amounts of unprocessed and less processed progastrin products [8,21,36,44,45,46]. In addition, gastrin peptides are less sulfated and the N-terminus of progastrin is less truncated. 

## 3. Extraintestinal Gastrin Expression

As previously described [37,38], the gastrin gene is also expressed in cells other than the antroduodenal G cells in the digestive tract. Quantitatively, these other cells normally contribute very little—if at all—to gastrin in plasma. This is because the level of expression is low because the secretion seems to serve local purposes and because the biosynthetic processing is cell-specific, i.e., different from that of the antroduodenal G cells. So far, we have seen extraantral and extrasmall intestinal expression of the gastrin gene in unidentified cells in the colon [11,47]; in endocrine cells in the fetal and neonatal pancreas [48,49,50]; in pituitary corticotrophs and melanotrophs [51,52,53]; in oxytocinergic hypothalamo-pituitary neurons [52,53]; in a few cerebellar and vagal neurons [54,55]; in the adrenal medulla [56]; in the bronchial mucosa [10]; in postmenopausal ovaria [12]; and in spermatogenic cells [57].

The function of gastrin synthesized outside the antroduodenal mucosa is unknown. However, one possibility is local paracrine regulation of growth. Another is that the peptides, although without a function in adults, are a relic of a more comprehensive fetal synthesis and function. The third possibility is that low cellular concentration reflects constitutive rather than regulated secretion. Although the extraantroduodenal synthesis of gastrin is without significance in the normal adult organism, the phenomenon is interesting from an oncofetal carcinogenetic and hence cancer diagnostic point of view. Thus, when carcinomas are developed from extraantral cells that normally express the gastrin gene at a low level, carcinomas may also express gastrin. However, cancer cell processing of progastrin is often tumor-specific. Colorectal cancers, for instance, cannot carboxyamidate gastrin precursors and, therefore, produce only progastrin-processing intermediates without effect on gastric acid secretion and growth [11].

## 4. Plasma Pattern and Concentration Levels of Gastrin in Hypergastrinemia

**Figure 4:**
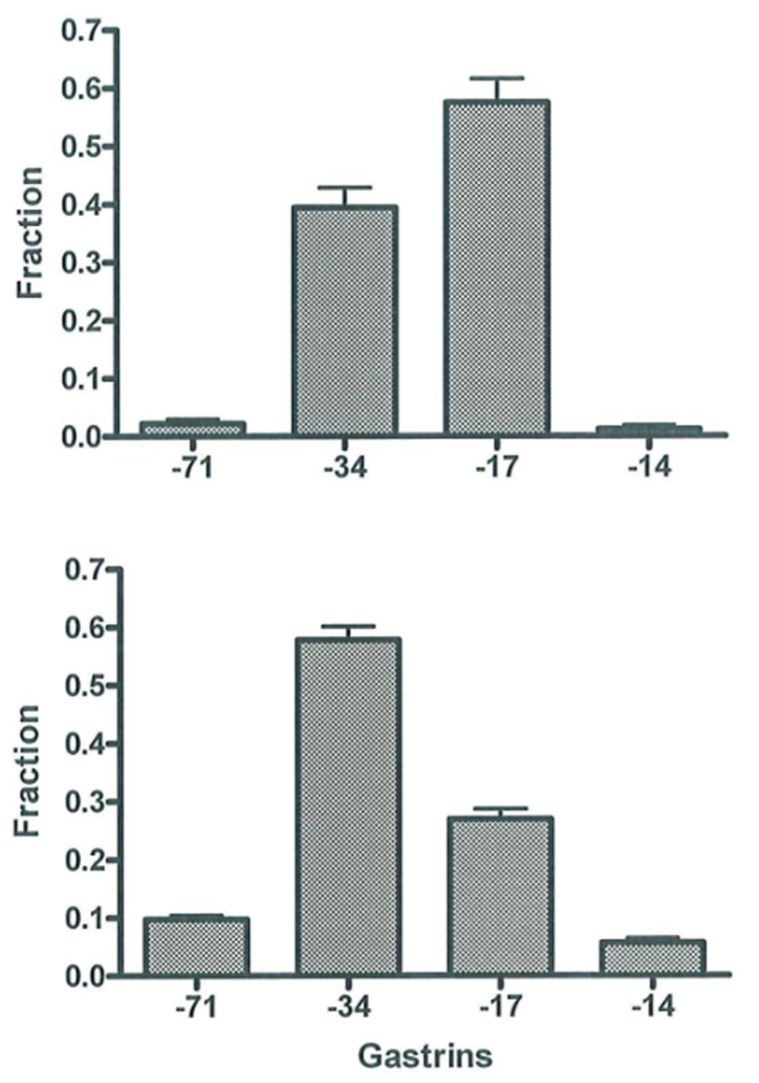
Molecular forms of gastrin (−71, −34, −17 and −14) in normogastrinemic serum or plasma (upper panel) and in hypergastrinemic plasma (lower panel). Data from [38] with permission.

Early studies showed—as also described before [38]—that plasma or serum from normal fasting subjects and normogastrinemic patients with duodenal ulcer contain almost similar concentrations of gastrin-34 and gastrin-17 and only little of other molecular forms [45], as shown in Figure 4 (upper panel). After a protein-rich meal, the initial rise in gastrin concentrations is due mainly to gastrin-17, but later gastrin-34 prevails [45]. This shift is in accordance with the content of gastrins in the secretory granules of antral G cells. Hence, normal G cells in a human contain, as mentioned, mostly gastrin-17 and only around 10% gastrin-34 [42,46]. However, since the metabolic clearance of, for instance, gastrin-34 from the circulation is 10-fold slower than that of gastrin-17 [58], peripheral plasma contains almost equal amounts of the two gastrins in the basal state and 30–45 min after larger meals [45].

The pattern of gastrin peptides in the circulation changes significantly due to permanently increased cellular secretion of gastrin as seen in antral G cells in achlorhydria. Hence, larger molecular forms, gastrin-71 and gastrin-34, predominate (Figure 4, lower panel). Accordingly, there is relatively little gastrin-17 in hypergastrinemic plasma (Figure 4). The mechanism behind the shift is considerably slower metabolic clearance of large gastrins [58,59], the effect of which becomes accentuated by hypersecretion. Moreover, due to permanent hypersecretion, progastrin products in secretory granules have, as mentioned above, less time for intracellular maturation before they are released to blood. Consequently, several granules may not achieve intragranular pH of 5.5, which is necessary for normal endoproteolytic cleavage by prohormone convertase 2 [39]. With reduced cleavage of processing intermediates, relatively less gastrin-17 is released. The combination of decreased cellular synthesis of gastrin-17 and slower clearance of gastrin-34, -52 and -71 from the circulation [58,59] results in the gastrin patterns in plasma as seen in Figure 4 (lower panel). Thus, the pathobiology of the biosynthesis in gastrin-producing cells and the different metabolic clearance rates of the different gastrins in the circulation explain the molecular shift in the pattern of gastrin peptides in peripheral plasma. This shift obviously necessitates immunoassays that measure the larger carboxyamidated forms of gastrin—both gastrin-34 and longer gastrins—with an affinity similar to that of gastrin-17 (toward which most antibodies are raised). Assays that measure only or mainly gastrin-17 result in false low concentrations (Table 2) [8,60]. Moreover, the discrepancy increases if mainly sulfated gastrin-17 is measured because the fraction of sulfated gastrins also decreases during hypersecretion [8,36].

Regarding concentration levels of circulating gastrins in hypergastrinemias, it is expedient to distinguish excessive hypergastrinemia from moderate hypergastrinemia at the level of 100 pmol/L (as suggested in Table 1). The conditions/diseases in these two categories differ. In this context, it should be noted that concentrations around 200 pmol/L are the level at which gastrins in plasma have the maximal effect on both gastric acid secretion and growth stimulation on ECL (enterochromaffin-like) cells in the stomach of both humans [61,62] and rats [63,64].

## 5. Measurement of Gastrin in Plasma

Accurate measurement of plasma or serum gastrin of sufficient reliability in both gastric hypo-, normo- and hypergastrinemias can be achieved using immunoassays. As mentioned before, the half-a-century-experience in our laboratory is primarily with the optimized in-house radioimmunoassay (RIA) technology, using high-titer and high-affinity antibodies with precisely defined epitope specificity. Thus, the antibodies have to be directed against the common *α*-amidated C-terminus (Figure 1 and Figure 3) so that they bind all bioactive gastrins with equimolar potency irrespective of the length of the N-terminal extension and degree of sulfation. Fortunately, such antibodies are fairly easy to raise, and they rarely cross-react with CCK peptides to any disturbing extent [65,66,67,68,69]. The RIA based on such antibodies measures hypergastrinemia accurately, both when all the gastrins circulate in increased concentrations (Figure 4; lower panel) and when only large molecular forms cause hypergastrinemia. Moreover, such gastrin RIA is simple and robust, and technically the analysis can be optimized to last only 1–2 h before the results are available. In addition, with high titers (>200,000), a high-affinity antiserum of adequate specificity can last for several decades. Thus, the rabbit antiserum still used for routine measurements of gastrin in our department was raised in 1970 [68].

## 6. *Helicobacter pylori*-Induced Hypergastrinemia

**Figure 5:**
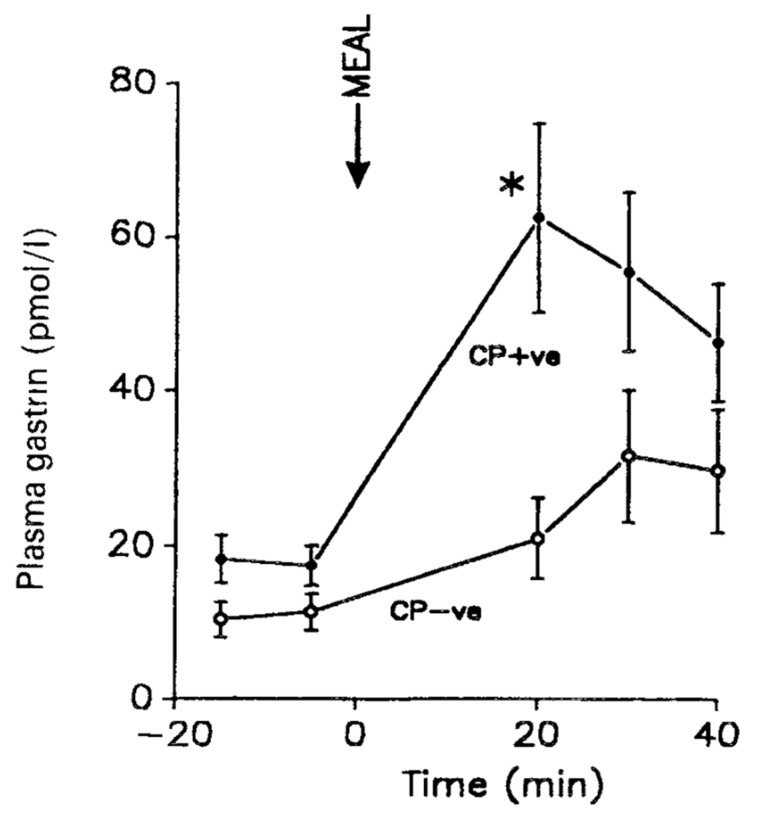
Meal-stimulated serum gastrin concentrations in duodenal ulcer patients with positive (CP+ve) or negative (CP–ve) biopsy urease tests for *Helicobacter pylori*. The arrow indicates the onset of the meal. From Levi et al. [18] with permission.

*Helicobacter pylori* (first named *Campolybacter pylori*) is a Gram-negative bacterium that often invades the stomach of young persons and causes chronic inflammation [1,2,24,70]. *Helicobacter pylori* survives the highly acidic environment in the stomach by production of ammonia, acid inhibitory factors and induction of inflammatory cytokines [24]. Most infected persons are without symptoms of the infection, but approximately 10% develop peptic ulcers, 1%—adenocarcinomas in the stomach [18,19,71]. A mainly antral infection is associated with hypersecretion of gastrin (Figure 5), subsequent increased acid secretion and duodenal ulcer disease, whereas infection in the fundic mucosa reduces gastric acid production and associates with adenocarcinoma development [21,24,72,73].

The mechanism of the hypergastrinemia induced by antral *Helicobacter pylori* infection appears to be an attack on antral somatostatin cells [22,23], which subsequently reduces the normal paracrine release of somatostatin that inhibits antral G cell secretion [18,19,21,74]. The ensuing hypergastrinemia is moderate, two to four times that of the gastrin secretion in healthy young subjects without *Helicobacter* infections [18,20,75]. Nevertheless, even such moderate hypergastrinemia appears essential in the subsequent gastric hyperacidity and fundamental in the pathogenesis of the duodenal ulcer disease [18,19,20,21].

Another aspect of the widespread *Helicobacter pylori*-induced hypergastrinemia is that the gastrin concentrations in plasma or serum from normal, healthy subjects reported in earlier studies [65,66,69] are too high because an unknown number of asymptomatic but infected persons are likely to have been included in the reference groups as normal control subjects. Consequently, the basal concentration levels of gastrin in uninfected, healthy fasting persons is 5–10 pmol/L [71] rather than the previously reported 20–25 pmol/L [65,66,69].

## 7. Proton-Pump Inhibitor-Induced Hypergastrinemia

The mean concentration of gastrins in plasma from healthy fasting subjects was—as mentioned above—earlier measured to be 20–25 pmol/L [45,69]. However, healthy subjects that are *Helicobacter*-negative display lower concentrations, often in the 5–10 pmol/L range [18,19,76]. In contrast, the plasma gastrin concentration varies considerably in fasting patients with chronic achlorhydria (as in pernicious anemia with preserved antral mucosa) from 100 to 2000 pmol/L [17,36]. Gastrin concentrations in fasting ulcer patients treated with PPI drugs are moderately increased, i.e., most levels reported are in between those of normal subjects and those of patients with severe long-term achlorhydria in pernicious anemia. The types and doses of PPI vary between the reports examined [25,26,27,28,29,30,31,32,33]. However, as shown in Table 3, there is generally a three–five-fold increase in the basal plasma concentrations of gastrin after daily oral doses of 40–80 mg PPI. It varies considerably, however, both individually and with the length of the period under drug administration. In a three-year study of patients refractory to H2-receptor antagonists, 11 of 106 fasting patients had serum gastrin concentrations above 250 pmol/L [30], including three with concentrations higher than 500 pmol/L. One cannot help but wonder whether the latter patients in fact might have harbored a gastrinoma [7,8].

## 8. Problem with Commercial Gastrin Kits

In comparison with in-house gastrin assays developed in university settings, commercial gastrin kits are, unfortunately, often problematic. As noted before [38], the original radioimmunoassays that measure gastrin concentrations in the circulation were developed in research laboratories at university departments and hospitals in different parts of the world [65,66,67,77]. The development was driven by curiosity about the role of gastrin in digestive physiology, pathophysiology and pharmacology [78,79,80]. Moreover, immunoassays also paved the way for the biochemical recognition that gastrin in plasma and tissue was not only gastrin-17 but the above-described mixture of progastrin-derived peptides of different lengths and amino acid derivations released in a cell-specific and sometimes disease-specific way. Accordingly, academic gastrin laboratories realized under which circumstances gastrin measurements are to be used for diagnosis of both excessive and moderate hypergastrinemic diseases. They also realized when further development and modifications of the immunoassay technology are expedient. The understanding, of course, required a clear distinction between analytical and diagnostic reliability and specificity of the assays employed. Thus, in the 1970s and 1980s, gastrin immunoassays were not only developed by scientists in university hospital laboratories, but the diagnostic use of gastrin assays was, to a large extent, also performed in university laboratories with a specific research interest in gastrin-related diseases and interpretation of the measurements.

In the recent decades, however, the research frontiers in gastroenterology have changed, and the research in gastrin biology and pathophysiology has been continued only in a smaller number of laboratories in Europe, North America, Japan and Australia. At the same time, the worldwide need for laboratory diagnosis of hypergastrinemic diseases has not diminished. Instead, it has opened a market for commercial gastrin kits during the past 25 years. The diagnosis of hypergastrinemic conditions is, therefore, to a large extent now based on commercial kits. In so far as these kits have the necessary specificity, accuracy and diagnostic meaningfulness, there is nothing wrong. However, the reliability requires not only insight into the assay technology. The biology and pathobiology of the gastrin peptide system and hypersecreting G cells also have to be known—not least of moderate hypergastrinemias as detailed above. Unfortunately, several kits display shortcomings in this respect because they only measure gastrin-17 (Table 2) [8,60] or have some other specificity problems [8].

## 9. Conclusions

Today, we know that most peptide hormones are complex and that a hormone exists in several different molecular forms which circulate in varying patterns in plasma. The patterns and concentration levels are modified by bacterial infections, drugs and tumor growth that affect the biogenesis of hormones. Consequently, the concentrations and the molecular pattern in plasma or serum change during the disease. Evidently, peptide hormone assays have to take these changes into account.

An example of such a complex hormone system is gastrin. An important issue with gastrin is moderate hypergastrinemias as seen in *Helicobacter pylori* infections and during long-term proton-pump inhibitor treatment as described above. Such moderate hypergastrinemias are significant risk factors for the development of gastric adenocarcinomas [81,82], possibly due to the interaction of *Helicobacter pylori* gastritis and acid inhibitory induced hypergastrinemia (for a recent review see [83]). Therefore, moderate hypergastrinemias have to be taken seriously. Consequently, the gastrin immunoassays used for diagnosis have to measure all the bioactive forms of gastrin in plasma in a reliable and accurate way.

## Figures and Tables

**Table 1:** Gastrinemias.

Category	Condition	Basal Plasma Concentration Levels (pmol/L)
Agastrinemia	Genetically modified animals	0
Hypogastrinemia	Antroduodenally resected patients Antro-mucosal atrophy patients	0–5
Normogastrinemia	Fasting normal subjects and mammals	5–15
Hypergastrinemia (I) (moderate)	*Helicobacter pylori*-infected subjects Proton-pump inhibitor-treated patients Early gastrinomas	20–100
Hypergastrinemia (II) (excessive)	Atrophic gastritis Gastrinomas with fulminant Zollinger–Ellison syndrome Gastric polyposis	>100

**Table 2:** Plasma concentrations of amidated gastrins versus gastrin-17 in consecutive gastrinoma patients.

Gastrinoma Patients	Amidated Gastrins (pmol/L)	Gastrin-17 (pmol/L)
1	475	10
2	235	42
3	270	90
4	82	15
5	57	24
6	60	26
7	305	6
Median	235	24

Data from [60]. Note that only patient No. 3 displays increased gastrin concentrations with the gastrin-17-specific immunoassay.

**Table 3:** Mean concentrations of gastrin in plasma before and after treatment with proton-pump inhibitors (PPIs).

Gastrin (pmol/L)		
before PPI	after PPI	Reference
19	115	[24]
14	27	[25]
6	20	[27]
12	39	[28]
–	46	[29]
6	17	[30]
7	34	[31]
10	35	[32]
